# The effect of mindfulness and self-compassion on compassion fatigue among nurses: a cross-sectional study

**DOI:** 10.1590/1980-220X-REEUSP-2026-0158en

**Published:** 2026-08-28

**Authors:** Yıldız Öztürk, Hilal Seki Öz

**Affiliations:** 1 Kırşehir Ahi Evran University, Faculty of Health Sciences, Department of Nursing, Kırşehir, Türkiye.

**Keywords:** Mindfulness, Self-Compassion, Compassion Fatigue, Nurses, Atenção Plena, Autocompaixão, Fadiga por Compaixão, Enfermeras y Enfermeros

## Abstract

**Objective:**

To examine the relationships between mindfulness, self-compassion, and compassion fatigue, and to determine the predictive roles of mindfulness and self-compassion on compassion fatigue among clinical nurses.

**Method:**

This cross-sectional correlational study included 220 nurses in a public hospital between March and June 2024. Data were collected via Personal Information Form, Mindful Attention Awareness Scale, Self-Compassion Scale-Short Form, and Compassion Fatigue Short Scale.

**Results:**

Mean scores were 61.78 ± 11.29 for mindfulness, 39.00 ± 6.87 for self-compassion, and 48.31 ± 20.28 for compassion fatigue. Mindfulness correlated positively with self-compassion (r = 0.33, p < 0.05) and negatively with compassion fatigue (r = -0.34, p < 0.05). Self-compassion was negatively associated with compassion fatigue (r = -0.40, p < 0.05). Multiple regression showed that mindfulness (β = -0.42) and self-compassion (β = -0.96) were significant independent predictors of compassion fatigue, jointly explaining 21.1% of the variance.

**Conclusion:**

Higher mindfulness and self-compassion predict lower compassion fatigue among clinical nurses. These modifiable psychological assets offer robust protection against emotional depletion. Integrating mindfulness and self-compassion into institutional strategies holds potential to optimize nursing well-being.

## RESUMO

**Objetivo:**

Examinar as relações entre atenção plena, autocompaixão e fadiga por compaixão e determinar os papéis preditivos da atenção plena e da autocompaixão na fadiga por compaixão entre enfermeiros clínicos.

**Método:**

Este estudo correlacional transversal incluiu 220 enfermeiros de um hospital público entre março e junho de 2024. Os dados foram coletados por meio de Formulário de Informações Pessoais, Escala de Atenção e Consciência Plena, Escala de Autocompaixão – Versão Curta e Escala Curta de Fadiga por Compaixão.

**Resultados:**

As pontuações médias foram de 61,78 ± 11,29 para atenção plena, 39,00 ± 6,87 para autocompaixão e 48,31 ± 20,28 para fadiga por compaixão. A atenção plena correlacionou-se positivamente com a autocompaixão (r = 0,33, p < 0,05) e negativamente com a fadiga por compaixão (r = -0,34, p < 0,05). A autocompaixão associou-se negativamente à fadiga por compaixão (r = -0,40, p < 0,05). A regressão múltipla mostrou que a atenção plena (β = -0,42) e a autocompaixão (β = -0,96) foram preditores independentes significativos da fadiga por compaixão, explicando conjuntamente 21,1% da variância.

**Conclusão:**

Níveis mais elevados de atenção plena e autocompaixão predizem menor fadiga por compaixão entre enfermeiros clínicos. Esses recursos psicológicos modificáveis oferecem uma proteção robusta contra o esgotamento emocional. A integração da atenção plena e da autocompaixão em estratégias institucionais tem o potencial de otimizar o bem-estar dos enfermeiros.

## INTRODUCTION

Clinical nursing requires continuous engagement with acute patient suffering and complex healthcare environments, which often expose professionals to substantial emotional depletion^(1)^. Prolonged exposure to these high-stress conditions without sufficient psychological mitigation frequently leads to the development of compassion fatigue, characterized by cumulative emotional and physical exhaustion^(2)^. Consequently, identifying internal safeguarding mechanisms is of paramount importance to preserve both nurse well-being and the quality of clinical care^(3)^. Mindfulness and self-compassion serve as critical personal resources in this context, effectively buffering against occupational distress while simultaneously cultivating sustainable psychological well-being among healthcare professionals^(4)^.

Mindfulness is defined as the ability to experience the present moment without judgment and has been shown to positively influence stress regulation, emotional flexibility, and cognitive functioning^(4)^. From an occupational health standpoint, mindfulness practices can strengthen nurses’ resilience against the psychosocial demands of intensive work environments, lessen the impact of workplace stressors, and enhance professional fulfillment^(5,6)^. Recent evidence suggests that mindfulness-based approaches may reduce compassion fatigue, burnout, and secondary traumatic stress while improving resilience and professional quality of life among nurses and healthcare professionals^(7,8,9)^. For these reasons, mindfulness is considered a protective factor that empowers nurses at the individual level while simultaneously contributing to broader organizational health and safety.

Self-compassion, which is regarded as one of the core dimensions of mindfulness, refers to adopting a kind and supportive stance toward oneself during moments of inadequacy or strain^(4)^. By enhancing individuals’ ability to regulate their emotions, self-compassion can mitigate the destructive impact of organizational stress and act as a psychological buffer against burnout^(7,10)^. Prior research indicates that nurses with higher self-compassion experience lower levels of anxiety and distress, subsequently leading to enhanced work performance and improved quality of patient care^(11)^. Taken together, these findings suggest that cultivating self-compassion alongside mindfulness may constitute a vital strategy for mitigating compassion fatigue in clinical nursing practice.

Compassion fatigue refers to a form of emotional depletion characterized by reduced empathy, exhaustion, and psychological strain that develops when healthcare workers are repeatedly confronted with distressing or traumatic patient situations^(12)^. Rising levels of compassion fatigue have been linked to adverse institutional outcomes such as workplace errors, threats to patient safety, and increased staff turnover, which has led to its classification as a critical occupational health risk^(13)^. Recent reviews have emphasized that nurses working in high-intensity clinical settings, particularly intensive care units, are especially vulnerable to compassion fatigue because of repeated exposure to critically ill patients, traumatic clinical events, ethical dilemmas, and high emotional demands^(14,15)^. Lowering compassion fatigue among nurses is vital for their personal well-being while remaining intrinsically linked to the long-term quality and stability of care delivery^(13,16)^.

Accumulated evidence indicates that interventions targeting mindfulness and self-compassion can reduce stress, anxiety, and burnout in healthcare professionals^(17,18)^. Although recent studies have increasingly examined mindfulness-based interventions and compassion fatigue among nurses, the relationships between mindfulness, self-compassion, and compassion fatigue in clinical nursing populations remain insufficiently explored, particularly within cross-sectional predictive models and non-Western healthcare contexts^(7,19)^. This gap underscores the need to identify new protective models for managing psychosocial risks in nursing practice. In line with this need, the present study aims to examine the relationships between mindfulness, self-compassion, and compassion fatigue, and to determine the predictive roles of mindfulness and self-compassion on compassion fatigue among clinical nurses. The findings are expected to provide empirical evidence that may inform in-service education, support nurses’ professional well-being, and guide sustainable occupational health policies^(20)^.

## METHOD

### Study Design

This study employed a cross-sectional correlational design to evaluate the relationships between mindfulness, self-compassion, and compassion fatigue, and to determine the predictive roles of mindfulness and self-compassion on compassion fatigue among clinical nurses.

### Study Setting and Participants

The study was conducted at Adana 5 Ocak State Hospital, a 200-bed public healthcare institution, between March and June 2024. The target population comprised all nurses actively employed in clinical units at the hospital (N = 232). A non-probability convenience sampling approach was utilized to recruit participants from the accessible population, aiming for a total population census. The inclusion criteria required being an actively working nurse with at least six months of professional experience at the institution. Administrative prerequisites, such as voluntary participation and the completion of all study forms, were fundamentally required for inclusion. Of the 232 eligible professionals, 220 met the criteria and fully completed the questionnaires, corresponding to an exceptionally high response rate of 94.8%. Nurses who declined participation, were on medical or annual leave during the data collection period, or submitted incomplete forms were excluded from the final sample.

### Data Collection

Data collection was executed between March and June 2024 through the face-to-face administration of paper-based questionnaires by the researchers. Nurses were approached during relatively low-intensity shift periods to minimize disruption to clinical workflows, and were comprehensively briefed regarding the study’s aims and procedures. A pilot application was conducted with 10 nurses to evaluate the clarity and comprehensibility of the items; since no structural modifications were required, these pilot data were excluded from the final analysis. Each questionnaire package required approximately 15–20 minutes to complete. The first author’s employment at the study institution facilitated seamless coordination of the data collection process and enhanced communication with participants, which fundamentally contributed to the high response rate.

### Instruments

*Personal Information Form:* Sociodemographic and occupational characteristics were assessed using a 16-item questionnaire developed by the researchers, capturing variables such as age, gender, professional experience, clinical unit, job satisfaction, exercise habits, and prior mindfulness training.

*Mindful Attention Awareness Scale (MAAS):* Originally developed by Brown and Ryan (2003) and adapted into Turkish by Özyeşil et al.^[21]^, the MAAS is a 15-item uni-dimensional scale that measures the frequency of mindful states in daily life. Items are rated on a six-point Likert scale ranging from 1 (almost always) to 6 (almost never), with higher cumulative scores reflecting greater levels of dispositional mindfulness. The Turkish version has demonstrated robust psychometric properties, with a reported Cronbach alpha coefficient of 0.80^(21)^.

*Self-Compassion Scale – Short Form (SCS-SF):* Developed by Raes et al. (2011) as a short form of Neff’s original 26-item scale, and adapted into Turkish by Yıldırım and Sarı^(22)^, this 12-item instrument measures self-compassion across six core dimensions: self-kindness, self-judgment, common humanity, isolation, mindfulness, and over-identification. Responses are rated on a five-point Likert scale ranging from 1 (almost never) to 5 (almost always), with higher scores indicating a stronger self-compassionate stance. The Turkish adaptation demonstrated acceptable internal consistency, with a Cronbach alpha coefficient of 0.75.

*Compassion Fatigue Short Scale (CFSS):* Originally developed by Adams et al. (2006) and adapted into Turkish by Dinç and Ekinci^(23)^, the scale is a self-report instrument consisting of 13 items evaluating two subdimensions: secondary traumatic stress and occupational burnout. Items are rated on a 10-point Likert response format ranging from 1 (rarely/never) to 10 (very often), where higher scores indicate greater severity of compassion fatigue. The Turkish validation reported Cronbach alpha coefficients for the subdimensions ranging between 0.80 and 0.90, indicating good internal consistency.

### Data Analysis

Statistical analyses were performed using SPSS version 25.0 (IBM Corp., Armonk, NY, USA). Descriptive statistics for categorical variables were expressed as absolute and relative frequencies formatted as n (%), while continuous variables were presented as means and standard deviations (M ± SD). Normality assumptions were verified using skewness and kurtosis indices within the acceptable range of ± 3. Group differences in sociodemographic and occupational characteristics were examined using independent samples t-tests and one-way ANOVA with Bonferroni post-hoc corrections where appropriate. Bivariate relationships among the primary constructs were assessed using Pearson correlation analysis. Finally, a multiple linear regression model was constructed to evaluate the predictive power of mindfulness and self-compassion on compassion fatigue. Statistical significance for all statistical models was established at p < 0.050.

### Ethical Considerations

Ethical approval was officially obtained from the Scientific Research and Publication Ethics Committee of Kırşehir Ahi Evran University (Decision No: 2023/11/24; Date: 21.12.2023). Institutional permission was subsequently secured from the Adana Provincial Health Directorate and Adana 5 Ocak State Hospital (Decision No: E-11289099-050.04-237875941; Date: 27.02.2024). Written and verbal informed consent was obtained from all participants prior to data gathering. The study was conducted strictly in accordance with the ethical principles delineated in the Declaration of Helsinki.

## RESULTS

The sociodemographic and occupational profile of the sample revealed that the cohort was predominantly female [181 (82.3%)] and held a university degree [196 (89.1%)]. The mean age of the participants was 29.16 ± 6.93 years, with a substantial proportion of the sample under 30 years of age [151 (68.6%)]. Regarding marital status and perceived financial standing, most nurses were single [149 (67.7%)] and reported a moderate economic status [166 (75.5%)]. Regarding health-related behaviors and clinical history, 49 (22.3%) participants smoked, 33 (15.0%) consumed alcohol, and the vast majority [205 (93.2%)] reported no chronic illnesses. The mean duration of professional experience was 6.33 ± 7.14 years, with 134 (60.9%) nurses having less than six years of tenure. Furthermore, 146 (66.4%) nurses stated that they had chosen the profession willingly, 145 (65.9%) reported enjoying their occupational role, and 148 (67.3%) worked rotating shifts. Regular physical exercise was reported by only 65 (29.5%) participants.

As presented in Table 1, the mean mindfulness score of the participants was 61.78 ± 11.29, indicating dispositional mindfulness levels well above the scale midpoint. Similarly, the mean self-compassion score was 39.00 ± 6.87, reflecting relatively high levels of self-compassion within the cohort. In contrast, the mean compassion fatigue score was 48.31 ± 20.28, suggesting comparatively low levels of compassion fatigue among the examined nursing population.

**Table 1:** Descriptive statistics of study variables – Kırşehir, Turkey, 2024.

Variables	Min	Max	Mean ± SD	Cronbach’s α
Mindfulness Scale	22.00	86.00	61.78 ± 11.29	0.844
Self-Compassion Scale	20.00	57.00	39.00 ± 6.87	0.799
Secondary Trauma Subscale	5.00	41.00	17.21 ± 7.90	0.722
Occupational Burnout Subscale	8.00	70.00	31.10 ± 14.04	0.833
Compassion Fatigue Scale (Total)	13.00	105.00	48.31 ± 20.28	0.873

As detailed in Table 2, group comparisons revealed several statistically significant variations across occupational and health-related characteristics. Nurses diagnosed with chronic illnesses exhibited significantly higher self-compassion scores than those without any chronic conditions (p < 0.050). Regarding professional attitudes, participants who reported enjoying their occupational role demonstrated markedly elevated mindfulness scores (p < 0.050) alongside substantially lower compassion fatigue levels (p < 0.050). Furthermore, compassion fatigue severity was found to be significantly higher among nurses who consumed alcohol (p < 0.050). Finally, clinical unit placement exerted a significant impact on compassion fatigue, with nurses working in high-intensity intensive care settings experiencing significantly higher levels of strain compared to their peers in other units (p < 0.050).

**Table 2:** Comparison of variables by demographics – Kırşehir, Turkey, 2024.

Variables			Mindfulness			Self-compassion			Compassion fatigue	
			$\bar{\text{X}}$ ± SD	Test/p		$\bar{\text{X}}$ ± SD	Test/p		$\bar{\text{X}}$ ± SD	Test/p
Gender	Male		63.23 ± 10.48	-0.881 0.378		39.49 ± 5.57	-0.492 0.623		45.33 ± 21.29	-1.010 0.314
	Female		61.47 ± 11.46			38.89 ± 7.12			48.95 ± 20.06	
Age	<30		62.50 ± 10.21	1.395 0.165		38.94 ± 7.14	-0.175 0.861		49.17 ± 20.19	0.933 0.352
	≥ 30		60.22 ± 13.30			39.12 ± 6.27			46.42 ± 20.51	
Marital status	Single		61.93 ± 10.25	0.861 0.424		38.89 ± 6.78	0.073 0.930		49.19 ± 19.20	1.081 0.341
	Married		62.20 ± 13.96			39.17 ± 7.18			47.62 ± 23.49	
	Divorced		57.45 ± 9.93			39.55 ± 6.88			40.09 ± 14.44	
Economical status	Good		62.34 ± 10.50	0.949 0.389		40.05 ± 7.21	1.018 0.362		44.21 ± 19.33	1.112 0.331
	Moderate		62.01 ± 11.39			38.62 ± 6.76			49.44 ± 20.70	
	Poor		58.06 ± 12.08			40.38 ± 7.11			46.31 ± 17.58	
Smoking	No		62.05 ± 11.43	0.648 0.517		38.98 ± 6.76	-0.075 0.940		47.57 ± 19.20	-1.005 0.316
	Yes		60.86 ± 10.86			39.06 ± 7.29			50.88 ± 23.73	
Alcohol use	No		62.31 ± 11.49	1.661 0.099		39.12 ± 6.85	0.656 0.513		46.86 ± 19.84	-2.552 **0.011**
	Yes		58.79 ± 9.71			38.27 ± 7.00			56.52 ± 21.14	
Chronic disease	No		61.68 ± 11.41	-0.503 0.615		38.69 ± 6.73	-2.453 **0.015**		48.86 ± 20.24	1.489 0.138
	Yes		63.20 ± 9.73			43.13 ± 7.61			40.80 ± 19.99	
Professional experience	<6 year		62.45 ± 10.09	1.090 0.276		38.80 ± 7.05	-0.531 0.597		47.64 ± 19.69	-0.608 0.544
	>6 year		60.74 ± 12.93			39.30 ± 6.60			49.35 ± 21.26	
Job liking	No		58.75 ± 11.57	-2.900 **0.004**		37.88 ± 6.38	-1.746 0.083		59.35 ± 18.79	6.296 **0.000**
	Yes		63.35 ± 10.85			39.57 ± 7.06			42.60 ± 18.65	
Clinical unit	Emergency^1^		61.53 ± 11.00	0.067 0.985		38.07 ± 6.70	1.837 0.108		50.02 ± 18.35	3.700 **0.002** 2 > 3 2 > 4
	Critical care^2^		62.86 ± 10.32			38.18 ± 5.84			56.39 ± 22.06	
	Clinic^3^		60.54 ± 11.96			41.00 ± 6.60			40.04 ± 18.72	
	Operating room^4^		60.89 ± 12.90			42.26 ± 6.90			44.37 ± 23.35	
	Inpatient Unit^5^		62.06 ± 11.26			38.56 ± 7.88			47.63 ± 18.12	
	Delivery Room^6^		62.07 ± 8.25			37.93 ± 6.85			43.67 ± 18.09	

Independent t-test, one-way ANOVA with Bonferroni correction; p < 0.050.

Bivariate correlation analysis showed that mindfulness correlated positively with self-compassion (r = 0.33, p < 0.05) and negatively with compassion fatigue (r = -0.344, p < 0.001). In a similar pattern, self-compassion exhibited robust negative correlations with secondary traumatic stress (r = -0.341, p < 0.001), occupational burnout (r = -0.390, p < 0.001), and total compassion fatigue scores (r = -0.403, p < 0.001), as summarized in Table 3.

**Table 3:** Correlations among variables – Kırşehir, Turkey, 2024.

Variables		1	2	3	4	5
Mindfulness Scale (1)	r	1.000	0.337	-0.271	-0.345	-0.344
	p	–	0.000*	0.000*	0.000*	0.000*
Self-Compassion Scale (2)	r		1.000	-0.341	-0.390	-0.403
	p		–	0.000*	0.000*	0.000*
Secondary Trauma Subscale (3)	r			1.000	0.685	0.863
	p			–	0.000*	0.000*
Occupational Burnout Subscale (4)	r				1.000	0.959
	p				–	0.000*
Compassion Fatigue Scale (5)	r					1.000
	p					–

Pearson correlation test, *p < 0.001.

Multiple linear regression analyses indicated that mindfulness significantly predicted lower compassion fatigue scores (β= -0.618, p < 0.001), explaining 11.8% of the variance. Self-compassion also emerged as a significant negative predictor of compassion fatigue (β = -1.190, p < 0.001), accounting for 16.2% of the variance. When both variables were simultaneously entered into the model, mindfulness (β = -0.422, p < 0.001) and self-compassion (β = -0.957, p < 0.001) remained significant independent predictors, jointly explaining 21.1% of the total variance in compassion fatigue, as shown in Table 4.

**Table 4:** Regression analysis results – Kırşehir, Turkey, 2024.

Model	Predictor	β (unstandardized)	Std. error	β (standardized)	t	p	F	p (model)	*R* ^2^
1	Constant	86.485	7.176	–	12.052	0.000	29.243	0.000	0.118
	Mindfulness	-0.618	0.114	-0.344	-5.408	0.000			
2	Constant	94.731	7.249	–	13.067	0.000	42.272	0.000	0.162
	Self-compassion	-1.190	0.183	-0.403	-6.502	0.000			
3	Constant	111.683	8.431	–	13.246	0.000	29.066	0.000	0.211
	Mindfulness	-0.422	0.115	-0.235	-3.667	0.000			
	Self-compassion	-0.957	0.189	-0.324	-5.059	0.000			

Linear regression analysis, p < 0.050.

## DISCUSSION

This study investigated the predictive and relational dynamics of mindfulness and self-compassion on compassion fatigue among clinical nurses. Our primary empirical findings revealed that higher levels of dispositional mindfulness and self-compassion are linked to mitigated levels of compassion fatigue, with both psychological constructs emerging as significant negative predictors in multivariate models. In clinical nursing, compassion fatigue represents a profound occupational health hazard, precipitated by unrelenting exposure to patient suffering, clinical crises, and secondary traumatic stress^(12,13)^. Recent occupational health literature confirms that mindfulness-based psychological resources provide a critical cognitive framework that enables healthcare professionals to process emotional burdens and chronic workplace stressors more effectively^(7,8)^. By fostering an objective, non-judgmental awareness of the present moment, mindfulness prevents nurses from over-identifying with traumatic clinical events, thereby interrupting the cognitive trajectory that leads from empathetic engagement to emotional depletion. Within high-acuity healthcare settings, internal resources that fortify cognitive flexibility, emotional regulation, and adaptive coping mechanisms are vital, not only for preserving the psychological well-being of the nursing workforce but also for sustaining the quality, safety, and stability of patient care delivery.

Nurses exhibiting elevated mindfulness capacities appear better equipped to modulate cognitive-emotional responses and sustain psychological equilibrium during high-stress clinical encounters. This capacity aligns with the conceptualization of mindfulness as the intentional, non-judgmental regulation of attention toward immediate experiences^(4)^. Empirical evidence indicates that heightened mindfulness fosters enhanced emotional granularity, attentional control, and cognitive flexibility, which collectively shield healthcare professionals from becoming emotionally overwhelmed by patient distress^(17,18)^. In congruence with our data, recent investigations confirm that superior mindfulness levels correspond directly to attenuated compassion fatigue across diverse nursing cohorts^(7)^. Moreover, structured mindfulness-based interventions have consistently demonstrated efficacy in reducing professional burnout, secondary traumatic stress, and compassion fatigue, while concurrently bolstering resilience and professional quality of life within the healthcare workforce^(8,9)^. This institutional utility is further corroborated by recent clinical trials among oncology nurses, where targeted mindfulness protocols significantly mitigated compassion fatigue while optimizing social support networks and baseline resilience^(19)^. Our finding of a robust negative association between mindfulness and compassion fatigue reinforces the paradigm that present-moment awareness serves as a vital, active psychological countermeasure within emotionally exhausting care environments.

An intriguing finding of the present study was that nurses diagnosed with chronic illnesses reported significantly higher self-compassion scores compared to their healthy peers. A plausible explanation for this phenomenon is that navigating a chronic health condition intrinsically heightens individuals’ awareness of personal vulnerability, subsequently compelling them to adopt more supportive, compassionate self-attitudes as a vital coping mechanism over time. Empirically, managing long-term health challenges has been shown to foster advanced emotional regulation, cognitive restructuring, and intensive self-care awareness, allowing clinical professionals to treat their own limitations with greater kindness rather than harsh self-judgment^(11,14,18)^. Furthermore, our data indicated that nurses who reported enjoying their profession demonstrated markedly elevated mindfulness levels alongside minimized compassion fatigue scores^(14,15)^. This dual association underscores a reciprocal, symbiotic dynamic between occupational satisfaction and psychological well-being. Positive professional engagement appears to enrich emotional coping reservoirs, whereas psychologically resilient individuals are concurrently more predisposed to derive deeper meaning and satisfaction from their clinical roles.

Self-compassion represents an adaptive emotional regulation strategy that enables individuals to respond to personal difficulties with kindness, acceptance, and minimized self-criticism. Empirical evidence has consistently linked heightened self-compassion with reduced levels of stress, anxiety, burnout, and emotional exhaustion among healthcare professionals^(7,8,10)^. In emotionally demanding occupations such as clinical nursing, self-compassion functions as a vital internal buffer, helping professionals tolerate acute emotional burdens more effectively while mitigating the internalization of workplace-related distress^(24)^. Specifically, by counteracting harsh self-judgment during perceived clinical failures or systemic challenges, self-compassion prevents empathetic distress from deteriorating into chronic emotional depletion. In alignment with these insights, nurses in the present study who exhibited higher self-compassion scores also reported significantly lower levels of compassion fatigue. This finding strongly reinforces the contemporary paradigm recognizing self-compassion as a malleable, dynamic psychological resource that can actively fortify emotional resilience, promote psychological safety, and preserve professional well-being across high-acuity nursing environments.

Compassion fatigue is increasingly recognized as a critical occupational health concern among nurses because of its detrimental effects on emotional well-being, quality of care, and workforce sustainability. In the present study, compassion fatigue levels were elevated among nurses working in intensive care units, those who had not willingly chosen the profession, and those reporting alcohol use. Intensive care environments are inherently characterized by repeated exposure to critically ill patients, complex ethical dilemmas, high emotional demands, and frequent encounters with suffering and death, all of which substantially increase vulnerability to emotional exhaustion and secondary traumatic stress^(13)^. Recent reviews have similarly emphasized that workload intensity, systemic staffing shortages, insufficient managerial support, and cumulative traumatic exposure are major contributors to compassion fatigue and professional burnout among intensive care staff^(14,15,24)^. Furthermore, maladaptive coping behaviors, such as alcohol consumption, and low professional commitment may undermine psychological resilience, thereby accelerating the progression of compassion fatigue over time. These findings emphasize that compassion fatigue extends beyond an individual psychological vulnerability; rather, it represents a multifaceted occupational health hazard shaped by the complex interplay between personal characteristics and systemic workplace factors.

The correlation and regression analyses demonstrated that both mindfulness and self-compassion were significantly associated with lower compassion fatigue, emerging as robust independent predictors in the final multivariate model. These outcomes align closely with established occupational frameworks reporting that mindfulness and self-compassion systematically mitigate burnout, emotional exhaustion, and secondary traumatic stress among frontline healthcare professionals^(7,10)^. Recent empirical evidence has similarly validated that superior mindfulness capacities correspond directly to diminished compassion fatigue across diverse nursing workforces^(7,11,14,25)^. Mechanistically, our predictive model suggests that nurses who maintain deliberate attention toward present-moment experiences while simultaneously fostering compassionate self-attitudes are substantially better equipped to navigate emotionally exhausting clinical demands. This phenomenon is highly consistent with contemporary stress-reduction frameworks emphasizing the dual importance of emotional regulation and cognitive flexibility; within these models, mindfulness facilitates the cognitive awareness and acceptance of distressing internal states, whereas self-compassion promotes a non-judgmental, adaptive response to acute occupational stressors ^(4,7)^. Concurrently, these psychological resources function in a highly complementary, synergistic manner, effectively replenishing emotional reserves and strengthening long-term professional resilience among clinical nurses.

According to the multiple linear regression models, mindfulness and self-compassion jointly accounted for 21.1% of the variance in compassion fatigue. While this predictive power underscores the critical importance of individual psychological resources, the remaining variance implicitly demonstrates that compassion fatigue is a highly multidimensional phenomenon governed by external organizational and environmental dynamics. Empirical evidence confirms that structural factors, such as excessive workload, inadequate staffing conditions, leadership styles, perceived organizational support, and the institutional ethical climate, substantially dictate the trajectory of compassion fatigue and professional burnout among nursing staff^(13,14,15,26)^. Future research should therefore systematically incorporate these institutional variables to construct more integrated, comprehensive explanatory models. Furthermore, longitudinal and intervention-based designs are warranted to elucidate the precise temporal and causal pathways underlying these interactions. Recent evidence indicates that structured mindfulness-based protocols represent feasible, scalable, and highly reproducible strategies for fortifying psychological resilience, optimizing professional quality of life, and securing comprehensive occupational well-being across modern healthcare delivery systems^(9,19,27,28)^.

## CONCLUSION

The findings of this study underscore that mindfulness and self-compassion serve as pivotal psychosocial assets that actively predict lower levels of compassion fatigue among clinical nurses. By reinforcing emotional regulation and self-directed kindness, these modifiable psychological resources offer robust protection against the emotional depletion inherent in high-acuity care settings. Integrating mindfulness and self-compassion-based protocols into institutional healthcare strategies holds the potential to simultaneously optimize nursing well-being and strengthen organizational sustainability. Future research should leverage longitudinal designs to track the long-term efficacy of these interventions and systematically investigate their synergistic interplay with systemic organizational variables such as workload, staffing ratios, and institutional climates.

### Strengths and Limitations

A primary strength of this investigation lies in its targeted evaluation of internal, modifiable psychological constructs, specifically mindfulness and self-compassion, as strategic countermeasures against compassion fatigue in the nursing workforce. Furthermore, the utilization of psychometrically validated instruments and the realization of an exceptionally high institutional response rate among actively practicing clinical nurses enhance the empirical robustness of our models.

Nevertheless, several limitations must be acknowledged. The cross-sectional nature of the study strictly precludes any definitive causal interpretations regarding the observed trajectories. Additionally, the omission of structural organizational covariates, such as objective workload metrics and managerial support systems, may have constrained the explanatory boundaries of the regression model. Finally, because the study’s data were gathered from a single public healthcare facility using a non-probability convenience sampling approach, and given the demographic predominance of relatively young, early-career professionals within the sample, caution must be exercised when generalizing these conclusions across broader or culturally diverse nursing populations.

### Implications for Practice

Our empirical evidence indicates that cultivating mindfulness and self-compassion provides an effective, proactive pathway to mitigate compassion fatigue and reinforce emotional resilience among frontline nurses. From an institutional perspective, embedding structured mindfulness training and self-compassion workshops into formal workplace well-being frameworks offers a practical, scalable mechanism to preemptively address occupational distress. Nurse managers and healthcare administrators are strongly encouraged to integrate these psychological competencies directly into newly hired nurse orientation protocols, ongoing in-service educational curricula, and professional staff support systems. At the macro-policy level, recognizing internal psychosocial fortifiers as essential components of comprehensive occupational health and safety strategies is vital for stabilizing the nursing workforce, reducing turnover intent, and ultimately elevating the safety and quality of patient care delivery.

## Data Availability

The data underlying this article will be shared on reasonable request to the corresponding author.
