# Supplementary material for: The effect of mindfulness and self-compassion on compassion fatigue among nurses: a cross-sectional study
Source: Rev Esc Enferm USP. 2026 Aug 28;60:e20260158. doi: 10.1590/1980-220X-REEUSP-2026-0158en (PMC13551601; doi:10.1590/1980-220X-REEUSP-2026-0158en)
Supplement: Translation

# Translation: El efecto de la atención plena (mindfulness) y la autocompasión en la fatiga por compasión entre el personal de enfermería: un estudio transversal

**DOI:** 10.1590/1980-220X-REEUSP-2026-0158es

**Authors:** Yıldız Öztürk, Hilal Seki Öz

**Affiliations:** 1 Kırşehir Ahi Evran University, Faculty of Health Sciences, Department of Nursing, Kırşehir, Türkiye.

**Keywords:** Atención Plena, Autocompasión, Desgaste por Empatía, Enfermeiras e Enfermeiros

## Abstract

**Objetivo:**

Examinar las relaciones entre la atención plena, la autocompasión y la fatiga por compasión, y determinar el papel predictivo de la atención plena y la autocompasión en la fatiga por compasión entre el personal de enfermería clínica.

**Método:**

Este estudio correlacional transversal incluyó a 220 enfermeras de un hospital público entre marzo y junio de 2024. Los datos se recolectaron mediante un formulario de información personal, la Escala de Conciencia de Atención Plena, la Escala de Autocompasión (versión corta) y la Escala Corta de Fatiga por Compasión.

**Resultados:**

Las puntuaciones promedio fueron de 61,78 ± 11,29 para la atención plena, 39,00 ± 6,87 para la autocompasión y 48,31 ± 20,28 para la fatiga por compasión. La atención plena se correlacionó positivamente con la autocompasión (r = 0,33; p < 0,05) y negativamente con la fatiga por compasión (r = -0,34; p < 0,05). La autocompasión se asoció negativamente con la fatiga por compasión (r = -0,40; p < 0,05). El análisis de regresión múltiple mostró que la atención plena (β = -0,42) y la autocompasión (β = -0,96) eran predictores independientes significativos de la fatiga por compasión, explicando conjuntamente el 21,1 % de la varianza.

**Conclusión:**

Un mayor nivel de atención plena y autocompasión predice una menor fatiga por compasión entre el personal de enfermería clínica. Estos recursos psicológicos modificables ofrecen una protección sólida contra el agotamiento emocional. La integración de la atención plena y la autocompasión en las estrategias institucionales tiene el potencial de optimizar el bienestar del personal de enfermería.

## INTRODUCCIÓN

La enfermería clínica requiere un compromiso continuo con el sufrimiento agudo de los pacientes y entornos de atención médica complejos, lo que a menudo expone a los profesionales a un agotamiento emocional considerable^(1)^. La exposición prolongada a estas condiciones de alto estrés sin una mitigación psicológica suficiente conduce con frecuencia al desarrollo de la fatiga por compasión, caracterizada por un agotamiento emocional y físico acumulativo^(2)^. En consecuencia, identificar mecanismos internos de protección es de suma importancia para preservar tanto el bienestar de las enfermeras como la calidad de la atención clínica^(3)^. La atención plena y la autocompasión sirven como recursos personales fundamentales en este contexto, amortiguando de manera efectiva el estrés laboral y, al mismo tiempo, cultivando un bienestar psicológico sostenible entre los profesionales de la salud^(4)^.

La atención plena (mindfulness) se define como la capacidad de experimentar el momento presente sin juzgar, y se ha demostrado que influye positivamente en la regulación del estrés, la flexibilidad emocional y el funcionamiento cognitivo^(4)^. Desde el punto de vista de la salud ocupacional, las prácticas de atención plena pueden fortalecer la resiliencia de las enfermeras frente a las exigencias psicosociales de los entornos de trabajo intensivos, disminuir el impacto de los factores estresantes del lugar de trabajo y mejorar la satisfacción profesional^(5,6)^. Evidencia reciente sugiere que los enfoques basados en la atención plena pueden reducir la fatiga por compasión, el agotamiento y el estrés traumático secundario, al tiempo que mejoran la resiliencia y la calidad de vida profesional entre el personal de enfermería y los profesionales de la salud^(7,8,9)^. Por estas razones, la atención plena se considera un factor protector que empodera al personal de enfermería a nivel individual y, al mismo tiempo, contribuye a la salud y seguridad organizacional en general.

La autocompasión, considerada una de las dimensiones centrales de la atención plena, se refiere a adoptar una postura amable y de apoyo hacia uno mismo en momentos de insuficiencia o tensión^(4)^. Al mejorar la capacidad de las personas para regular sus emociones, la autocompasión puede mitigar el impacto destructivo del estrés organizacional y actuar como un amortiguador psicológico contra el agotamiento^(7,10)^. Investigaciones previas indican que las enfermeras con mayor autocompasión experimentan niveles más bajos de ansiedad y angustia, lo que a su vez conduce a un mejor desempeño laboral y a una mejor calidad de la atención al paciente^(11)^. En conjunto, estos hallazgos sugieren que cultivar la autocompasión junto con la atención plena puede constituir una estrategia vital para mitigar la fatiga por compasión en la práctica clínica de la enfermería.

La fatiga por compasión se refiere a una forma de agotamiento emocional caracterizada por una disminución de la empatía, el agotamiento y la tensión psicológica que se desarrolla cuando los trabajadores de la salud se enfrentan repetidamente a situaciones angustiosas o traumáticas con los pacientes^(12)^. El aumento de los niveles de fatiga por compasión se ha relacionado con resultados institucionales adversos, tales como errores en el lugar de trabajo, amenazas a la seguridad de los pacientes y una mayor rotación del personal, lo que ha llevado a su clasificación como un riesgo crítico para la salud ocupacional^(13)^. Revisiones recientes han destacado que las enfermeras que trabajan en entornos clínicos de alta intensidad, particularmente en unidades de cuidados intensivos, son especialmente vulnerables a la fatiga por compasión debido a la exposición repetida a pacientes en estado crítico, eventos clínicos traumáticos, dilemas éticos y altas exigencias emocionales^(14,15)^. Reducir la fatiga por compasión entre las enfermeras es vital para su bienestar personal, al tiempo que está intrínsecamente vinculado a la calidad y estabilidad a largo plazo de la prestación de cuidados^(13,16)^.

La evidencia acumulada indica que las intervenciones centradas en la atención plena y la autocompasión pueden reducir el estrés, la ansiedad y el agotamiento en los profesionales de la salud^(17,18)^. Aunque estudios recientes han analizado cada vez más las intervenciones basadas en la atención plena y la fatiga por compasión entre el personal de enfermería, las relaciones entre la atención plena, la autocompasión y la fatiga por compasión en poblaciones clínicas de enfermería siguen sin explorarse lo suficiente, particularmente en modelos predictivos transversales y en contextos de atención médica no occidentales^(7,19)^. Esta brecha subraya la necesidad de identificar nuevos modelos de protección para gestionar los riesgos psicosociales en la práctica de la enfermería. En consonancia con esta necesidad, el presente estudio tiene como objetivo examinar las relaciones entre la atención plena, la autocompasión y la fatiga por compasión, y determinar el papel predictivo de la atención plena y la autocompasión sobre la fatiga por compasión entre el personal de enfermería clínica. Se espera que los hallazgos proporcionen evidencia empírica que pueda servir de base para la formación continua, apoyar el bienestar profesional de las enfermeras y orientar políticas sostenibles de salud ocupacional^(20)^.

## MÉTODO

### Diseño del Estudio

Este estudio empleó un diseño correlacional transversal para evaluar las relaciones entre la atención plena, la autocompasión y la fatiga por compasión, y para determinar el papel predictivo de la atención plena y la autocompasión sobre la fatiga por compasión entre las enfermeras clínicas.

### Entorno del Estudio y Participantes

El estudio se llevó a cabo en el Hospital Estatal 5 Ocak de Adana, una institución de salud pública con 200 camas, entre marzo y junio de 2024. La población objetivo estuvo compuesta por todas las enfermeras que trabajaban activamente en las unidades clínicas del hospital (N = 232). Se utilizó un enfoque de muestreo por conveniencia no probabilístico para reclutar participantes de la población accesible, con el objetivo de realizar un censo de la población total. Los criterios de inclusión exigían ser enfermero en activo con al menos seis meses de experiencia profesional en la institución. Los requisitos administrativos, como la participación voluntaria y la cumplimentación de todos los formularios del estudio, eran fundamentales para la inclusión. De los 232 profesionales elegibles, 220 cumplieron con los criterios y completaron íntegramente los cuestionarios, lo que corresponde a una tasa de respuesta excepcionalmente alta del 94,8 %. Se excluyeron de la muestra final las enfermeras que rechazaron participar, que se encontraban de baja médica o de vacaciones anuales durante el período de recolección de datos, o que presentaron formularios incompletos.

### Recolección de Datos

La recolección de datos se llevó a cabo entre marzo y junio de 2024 mediante la administración presencial de cuestionarios en papel por parte de los investigadores. Se abordó a las enfermeras durante turnos de intensidad relativamente baja para minimizar la interrupción de los flujos de trabajo clínicos, y se les informó exhaustivamente sobre los objetivos y procedimientos del estudio. Se llevó a cabo una prueba piloto con 10 enfermeras para evaluar la claridad y comprensibilidad de los ítems; dado que no se requirieron modificaciones estructurales, estos datos piloto se excluyeron del análisis final. Cada conjunto de cuestionarios requirió aproximadamente entre 15 y 20 minutos para completarse. El hecho de que el primer autor trabajara en la institución del estudio facilitó una coordinación fluida del proceso de recolección de datos y mejoró la comunicación con las participantes, lo que contribuyó fundamentalmente a la alta tasa de respuesta.

### Instrumentos

*Formulario de información personal:* Las características sociodemográficas y ocupacionales se evaluaron mediante un cuestionario de 16 ítems desarrollado por los investigadores, que abarcaba variables como la edad, el género, la experiencia profesional, la unidad clínica, la satisfacción laboral, los hábitos de ejercicio y la formación previa en atención plena.

*Escala de Conciencia de la Atención Plena (Mindful Attention Awareness Scal*e – *MAAS):* Desarrollada originalmente por Brown y Ryan (2003) y adaptada al turco por Özyeşil et al.^(21)^, la MAAS es una escala unidimensional de 15 ítems que mide la frecuencia de los estados de atención plena en la vida cotidiana. Los ítems se califican en una escala de Likert de seis puntos que va de 1 (casi siempre) a 6 (casi nunca), y las puntuaciones acumuladas más altas reflejan mayores niveles de atención plena disposicional. La versión en turco ha demostrado propiedades psicométricas sólidas, con un coeficiente alfa de Cronbach reportado de 0,80^(21)^.

*Escala de Autocompasión – Versión Corta (Self-Compassion Scale – Short Form* – *SCS-SF):* Desarrollada por Raes et al. (2011) como una versión abreviada de la escala original de 26 ítems de Neff, y adaptada al turco por Yıldırım y Sarı^(22)^, este instrumento de 12 ítems mide la autocompasión a través de seis dimensiones fundamentales: amabilidad hacia uno mismo, juicio hacia uno mismo, humanidad común, aislamiento, atención plena y sobreidentificación. Las respuestas se califican en una escala de Likert de cinco puntos que va de 1 (casi nunca) a 5 (casi siempre), donde las puntuaciones más altas indican una actitud de autocompasión más fuerte. La adaptación al turco demostró una consistencia interna aceptable, con un coeficiente alfa de Cronbach de 0,75.

*Escala Breve de Fatiga Compasiva (Compassion Fatigue Short Scale* – *CFSS):* Desarrollada originalmente por Adams et al. (2006) y adaptada al turco por Dinç y Ekinci^(23)^, la escala es un instrumento de autoinforme que consta de 13 ítems que evalúan dos subdimensiones: estrés traumático secundario y agotamiento laboral. Los ítems se califican en una escala de respuesta de Likert de 10 puntos que va de 1 (rara vez/nunca) a 10 (muy a menudo), donde las puntuaciones más altas indican una mayor gravedad de la fatiga por compasión. La validación en turco reportó coeficientes alfa de Cronbach para las subdimensiones que oscilaron entre 0,80 y 0,90, lo que indica una buena consistencia interna.

### Análisis de Datos

Los análisis estadísticos se realizaron con el programa SPSS versión 25.0 (IBM Corp., Armonk, NY, EE. UU.). Las estadísticas descriptivas para las variables categóricas se expresaron como frecuencias absolutas y relativas en formato n (%), mientras que las variables continuas se presentaron como medias y desviaciones estándar (M ± DE). Los supuestos de normalidad se verificaron mediante los índices de asimetría y curtosis, que se mantuvieron dentro del rango aceptable de ± 3. Las diferencias entre grupos en las características sociodemográficas y ocupacionales se examinaron mediante pruebas t para muestras independientes y ANOVA unidireccional con correcciones post hoc de Bonferroni cuando fue apropiado. Las relaciones bivariadas entre los constructos principales se evaluaron mediante el análisis de correlación de Pearson. Finalmente, se construyó un modelo de regresión lineal múltiple para evaluar el poder predictivo de la atención plena y la autocompasión sobre la fatiga por compasión. La significación estadística para todos los modelos estadísticos se estableció en p < 0,050.

### Consideraciones Éticas

Se obtuvo oficialmente la aprobación ética del Comité de Ética en Investigación Científica y Publicaciones de la Universidad Ahi Evran de Kırşehir (Decisión n.º: 2023/11/24; Fecha: 21 de diciembre de 2023). Posteriormente, se obtuvo el permiso institucional de la Dirección Provincial de Salud de Adana y del Hospital Estatal 5 Ocak de Adana (Decisión n.º: E-11289099-050.04-237875941; Fecha: 27 de febrero de 2024). Se obtuvo el consentimiento informado, tanto escrito como verbal, de todos los participantes antes de la recopilación de datos. El estudio se llevó a cabo estrictamente de acuerdo con los principios éticos establecidos en la Declaración de Helsinki.

## RESULTADOS

El perfil sociodemográfico y laboral de la muestra reveló que la cohorte estaba compuesta predominantemente por mujeres [181 (82,3 %)] y que la mayoría contaba con un título universitario [196 (89,1 %)]. La edad promedio de los participantes fue de 29,16 ± 6,93 años, con una proporción considerable de la muestra menor de 30 años [151 (68,6%)]. En cuanto al estado civil y la situación económica percibida, la mayoría de las enfermeras eran solteras [149 (67,7%)] y declararon tener una situación económica moderada [166 (75,5%)]. En cuanto a los hábitos relacionados con la salud y los antecedentes clínicos, 49 (22,3 %) participantes fumaban, 33 (15,0 %) consumían alcohol y la gran mayoría [205 (93,2 %)] declararon no padecer enfermedades crónicas. La duración promedio de la experiencia profesional fue de 6,33 ± 7,14 años, y 134 (60,9 %) enfermeras contaban con menos de seis años de antigüedad. Además, 146 (66,4 %) enfermeras afirmaron haber elegido la profesión por voluntad propia, 145 (65,9 %) indicaron que disfrutaban de su rol laboral y 148 (67,3 %) trabajaban en turnos rotativos. Solo 65 (29,5 %) participantes informaron realizar ejercicio físico regularmente.

Como se presenta en la Tabla 1, la puntuación media de atención plena de las participantes fue de 61,78 ± 11,29, lo que indica niveles de atención plena disposicional muy por encima del punto medio de la escala. De manera similar, la puntuación media de autocompasión fue de 39,00 ± 6,87, lo que refleja niveles relativamente altos de autocompasión dentro de la cohorte. Por el contrario, la puntuación media de fatiga por compasión fue de 48,31 ± 20,28, lo que sugiere niveles comparativamente bajos de fatiga por compasión entre la población de enfermeras examinada.

**Tabla 1:** Estadísticas descriptivas de las variables del estudio – Kırşehir, Turquía, 2024.

Variables	Mín.	Máx	Media ± DE	α de Cronbach
Escala de atención plena	22,00	86,00	61,78 ± 11,29	0,844
Escala de autocompasión	20,00	57,00	39,00 ± 6,87	0,799
Subescala de trauma secundario	5,00	41,00	17,21 ± 7,90	0,722
Subescala de agotamiento (burnout) laboral	8,00	70,00	31,10 ± 14,04	0,833
Escala de fatiga por compasión (total)	13,00	105,00	48,31 ± 20,28	0,873

Tal como se detalla en la Tabla 2, las comparaciones entre grupos revelaron varias variaciones estadísticamente significativas en función de las características profesionales y relacionadas con la salud. Las enfermeras diagnosticadas con enfermedades crónicas presentaron puntuaciones de autocompasión significativamente más altas que aquellas sin ninguna afección crónica (p < 0,050). En cuanto a las actitudes profesionales, las participantes que afirmaron disfrutar de su papel profesional mostraron puntuaciones de atención plena notablemente más elevadas (p < 0,050), junto con niveles de fatiga por compasión sustancialmente más bajos (p < 0,050). Además, se observó que la gravedad de la fatiga por compasión era significativamente mayor entre las enfermeras que consumían alcohol (p < 0,050). Por último, la unidad clínica en la que se trabajaba ejerció un impacto significativo en la fatiga por compasión, ya que las enfermeras que trabajaban en entornos de cuidados intensivos de alta intensidad experimentaban niveles de estrés significativamente más elevados en comparación con sus compañeras de otras unidades (p < 0,050).

**Tabla 2:** Comparación de variables según características demográficas – Kırşehir, Turquía, 2024.

Variables			Atención plena (mindfulness)			Autocompasión			Fatiga por compasión	
			$\bar{\text{X}}$ ± DE	Prueba/p		$\bar{\text{X}}$ ± DE	Prueba/p		$\bar{\text{X}}$ ± DE	Prueba/p
Género	Hombres		63,23 ± 10,48	-0,881 0,378		39,49 ± 5,57	-0,492 0,623		45,33 ± 21,29	-1,010 0,314
	Mujer		61,47 ± 11,46			38,89 ± 7,12			48,95 ± 20,06	
Edad	<30		62,50 ± 10,21	1,395 0,165		38,94 ± 7,14	-0,175 0,861		49,17 ± 20,19	0,933 0,352
	≥ 30		60,22 ± 13,30			39,12 ± 6,27			46,42 ± 20,51	
Estado civil	Soltero		61,93 ± 10,25	0,861 0,424		38,89 ± 6,78	0,073 0,930		49,19 ± 19,20	1,081 0,341
	Casado		62,20 ± 13,96			39,17 ± 7,18			47,62 ± 23,49	
	Divorciados		57,45 ± 9,93			39,55 ± 6,88			40,09 ± 14,44	
Situación económica	Buena		62,34 ± 10,50	0,949 0,389		40,05 ± 7,21	1,018 0,362		44,21 ± 19,33	1,112 0,331
	Moderado		62,01 ± 11,39			38,62 ± 6,76			49,44 ± 20,70	
	Deficiente		58,06 ± 12,08			40,38 ± 7,11			46,31 ± 17,58	
Tabaquismo	No		62,05 ± 11,43	0,648 0,517		38,98 ± 6,76	-0,075 0,940		47,57 ± 19,20	-1,005 0,316
	Sí		60,86 ± 10,86			39,06 ± 7,29			50,88 ± 23,73	
Consumo de alcohol	No		62,31 ± 11,49	1,661 0,099		39,12 ± 6,85	0,656 0,513		46,86 ± 19,84	-2,552 **0,011**
	Sí		58,79 ± 9,71			38,27 ± 7,00			56,52 ± 21,14	
Enfermedad crónica	No		61,68 ± 11,41	-0,503 0,615		38,69 ± 6,73	-2,453 **0,015**		48,86 ± 20,24	1,489 0,138
	Sí		63,20 ± 9,73			43,13 ± 7,61			40,80 ± 19,99	
Experiencia profesional	<6 años		62,45 ± 10,09	1,090 0,276		38,80 ± 7,05	-0,531 0,597		47,64 ± 19,69	-0,608 0,544
	>6 años		60,74 ± 12,93			39,30 ± 6,60			49,35 ± 21,26	
Satisfacción laboral	No		58,75 ± 11,57	-2,900 **0,004**		37,88 ± 6,38	-1,746 0,083		59,35 ± 18,79	6,296 **0,000**
	Sí		63,35 ± 10,85			39,57 ± 7,06			42,60 ± 18,65	
Unidad clínica	Urgencias^1^		61,53 ± 11,00	0,067 0,985		38,07 ± 6,70	1,837 0,108		50,02 ± 18,35	3,700 **0,002** 2 > 3 2 > 4
	Cuidados intensivos^2^		62,86 ± 10,32			38,18 ± 5,84			56,39 ± 22,06	
	Clínica^3^		60,54 ± 11,96			41,00 ± 6,60			40,04 ± 18,72	
	Quirófano^4^		60,89 ± 12,90			42,26 ± 6,90			44,37 ± 23,35	
	Unidad de hospitalización^5^		62,06 ± 11,26			38,56 ± 7,88			47,63 ± 18,12	
	Sala de partos^6^		62,07 ± 8,25			37,93 ± 6,85			43,67 ± 18,09	

Prueba t independiente, ANOVA unidireccional con corrección de Bonferroni; p < 0,050.

El análisis de correlación bivariado mostró que la atención plena se correlacionaba positivamente con la autocompasión (r = 0,33; p < 0,05) y negativamente con la fatiga por compasión (r = -0,344; p < 0,001). Siguiendo un patrón similar, la autocompasión mostró fuertes correlaciones negativas con el estrés traumático secundario (r = -0,341, p < 0,001), el agotamiento laboral (r = -0,390, p < 0,001) y las puntuaciones totales de fatiga por compasión (r = -0,403, p < 0,001), tal y como se resume en la Tabla 3.

**Tabla 3:** Correlaciones entre variables – Kırşehir, Turquía, 2024.

Variables		1	2	3	4	5
Escala de atención plena (1)	r	1,000	0,337	-0,271	-0,345	-0,344
	p	–	0,000*	0,000*	0,000*	0,000*
Escala de autocompasión (2)	r		1,000	-0,341	-0,390	-0,403
	p		–	0,000*	0,000*	0,000*
Subescala de trauma secundario (3)	r			1,000	0,685	0,863
	p			–	0,000*	0,000*
Subescala de agotamiento laboral (4)	r				1,000	0,959
	p				–	0,000*
Escala de fatiga por compasión (5)	r					1,000
	p					–

Prueba de correlación de Pearson

*p < 0,001.

Los análisis de regresión lineal múltiple indicaron que la atención plena predecía de forma significativa puntuaciones más bajas de fatiga por compasión (β = -0,618, p < 0,001), lo que explicaba el 11,8 % de la varianza. La autocompasión también se reveló como un predictor negativo significativo de la fatiga por compasión (β = -1,190, p < 0,001), representando el 16,2 % de la varianza. Cuando ambas variables se introdujeron simultáneamente en el modelo, la atención plena (β = -0,422, p < 0,001) y la autocompasión (β = -0,957, p < 0,001) siguieron siendo predictores independientes significativos, explicando conjuntamente el 21,1 % de la varianza total en la fatiga por compasión, tal y como se muestra en la Tabla 4.

**Tabla 4:** Resultados del análisis de regresión – Kırşehir, Turquía, 2024.

Modelo	Predictor	β (no estandarizado)	Error estándar	β (estandarizado)	t	p	F	p (modelo)	*R* ^2^
1	Constante	86,485	7,176	–	12,052	0,000	29,243	0,000	0,118
	Atención plena	-0,618	0,114	-0,344	-5,408	0,000			
2	Constante	94,731	7,249	–	13,067	0,000	42,272	0,000	0,162
	Autocompasión	-1,190	0,183	-0,403	-6,502	0,000			
3	Constante	111,683	8,431	–	13,246	0,000	29,066	0,000	0,211
	Atención plena	-0,422	0,115	-0,235	-3,667	0,000			
	Autocompasión	-0,957	0,189	-0,324	-5,059	0,000			

Análisis de regresión lineal, p < 0,050.

## DISCUSIÓN

Este estudio investigó la dinámica predictiva y relacional de la atención plena y la autocompasión en la fatiga por compasión entre el personal de enfermería clínica. Nuestros principales hallazgos empíricos revelaron que los niveles más altos de atención plena disposicional y autocompasión están relacionados con niveles más bajos de fatiga por compasión, y ambos constructos psicológicos se revelaron como predictores negativos significativos en los modelos multivariados. En la enfermería clínica, la fatiga por compasión representa un grave riesgo para la salud ocupacional, provocado por la exposición constante al sufrimiento de los pacientes, las crisis clínicas y el estrés traumático secundario^(12,13)^. La literatura reciente sobre salud ocupacional confirma que los recursos psicológicos basados en la atención plena proporcionan un marco cognitivo fundamental que permite a los profesionales de la salud procesar las cargas emocionales y los factores estresantes crónicos del lugar de trabajo de manera más eficaz^(7,8)^. Al fomentar una conciencia objetiva y sin juicios del momento presente, la atención plena evita que las enfermeras se identifiquen en exceso con los eventos clínicos traumáticos, interrumpiendo así la trayectoria cognitiva que conduce del compromiso empático al agotamiento emocional. En entornos de atención médica de alta complejidad, los recursos internos que fortalecen la flexibilidad cognitiva, la regulación emocional y los mecanismos de afrontamiento adaptativos son vitales, no solo para preservar el bienestar psicológico del personal de enfermería, sino también para mantener la calidad, la seguridad y la estabilidad de la prestación de cuidados a los pacientes.

Las enfermeras que muestran un alto nivel de atención plena parecen estar mejor preparadas para modular las respuestas cognitivo-emocionales y mantener el equilibrio psicológico durante situaciones clínicas de alto estrés. Esta capacidad concuerda con la conceptualización de la atención plena como la regulación intencional y sin juicios de la atención hacia las experiencias inmediatas^(4)^. La evidencia empírica indica que una mayor atención plena fomenta una mayor granularidad emocional, control atencional y flexibilidad cognitiva, lo cual, en conjunto, protege a los profesionales de la salud de sentirse abrumados emocionalmente por el sufrimiento de los pacientes^(17,18)^. En consonancia con nuestros datos, investigaciones recientes confirman que los niveles superiores de atención plena se corresponden directamente con una fatiga por compasión atenuada en diversas cohortes de enfermeras^(7)^. Además, las intervenciones estructuradas basadas en la atención plena han demostrado de manera consistente su eficacia para reducir el agotamiento profesional, el estrés traumático secundario y la fatiga por compasión, al tiempo que refuerzan la resiliencia y la calidad de vida profesional del personal de salud^(8,9)^. Esta utilidad institucional se ve corroborada además por ensayos clínicos recientes entre enfermeras oncológicas, en los que los protocolos específicos de atención plena mitigaron significativamente la fatiga por compasión al tiempo que optimizaron las redes de apoyo social y la resiliencia inicial^(19)^. Nuestro hallazgo de una sólida asociación negativa entre la atención plena y la fatiga por compasión refuerza el paradigma de que la conciencia del momento presente sirve como una contramedida psicológica vital y activa en entornos de atención emocionalmente agotadores.

Un hallazgo interesante del presente estudio fue que las enfermeras diagnosticadas con enfermedades crónicas reportaron puntuaciones de autocompasión significativamente más altas en comparación con sus colegas sanas. Una explicación plausible de este fenómeno es que lidiar con una afección de salud crónica aumenta intrínsecamente la conciencia de las personas sobre su propia vulnerabilidad, lo que posteriormente las impulsa a adoptar actitudes hacia sí mismas más solidarias y compasivas como un mecanismo vital de afrontamiento a lo largo del tiempo. Empíricamente, se ha demostrado que manejar desafíos de salud a largo plazo fomenta una regulación emocional avanzada, una reestructuración cognitiva y una conciencia intensiva del autocuidado, lo que permite a los profesionales clínicos tratar sus propias limitaciones con mayor amabilidad en lugar de un juicio severo hacia sí mismos^(11,14,18)^. Además, nuestros datos indicaron que las enfermeras que manifestaron disfrutar de su profesión mostraron niveles notablemente elevados de atención plena, junto con puntuaciones minimizadas de fatiga por compasión^(14,15)^. Esta doble asociación subraya una dinámica recíproca y simbiótica entre la satisfacción laboral y el bienestar psicológico. El compromiso profesional positivo parece enriquecer las reservas emocionales para afrontar las dificultades, mientras que las personas psicológicamente resilientes están, al mismo tiempo, más predispuestas a obtener un significado y una satisfacción más profundos de sus roles clínicos.

La autocompasión representa una estrategia adaptativa de regulación emocional que permite a las personas responder a las dificultades personales con amabilidad, aceptación y una autocrítica mínima. La evidencia empírica ha relacionado de manera consistente una mayor autocompasión con niveles reducidos de estrés, ansiedad, agotamiento y agotamiento emocional entre los profesionales de la salud^(7,8,10)^. En ocupaciones emocionalmente exigentes, como la enfermería clínica, la autocompasión funciona como un amortiguador interno vital, que ayuda a los profesionales a tolerar las cargas emocionales agudas de manera más eficaz, al tiempo que mitiga la internalización de la angustia relacionada con el lugar de trabajo^(24)^. Específicamente, al contrarrestar el juicio severo hacia uno mismo durante los fracasos clínicos percibidos o los desafíos sistémicos, la autocompasión evita que la angustia empática se convierta en un agotamiento emocional crónico. En consonancia con estas ideas, las enfermeras del presente estudio que mostraron puntuaciones más altas de autocompasión también reportaron niveles significativamente más bajos de fatiga por compasión. Este hallazgo refuerza firmemente el paradigma contemporáneo que reconoce la autocompasión como un recurso psicológico maleable y dinámico que puede fortalecer activamente la resiliencia emocional, promover la seguridad psicológica y preservar el bienestar profesional en entornos de enfermería de alta complejidad.

La fatiga por compasión se reconoce cada vez más como un problema crítico de salud ocupacional entre el personal de enfermería debido a sus efectos perjudiciales sobre el bienestar emocional, la calidad de la atención y la sostenibilidad de la fuerza laboral. En el presente estudio, los niveles de fatiga por compasión fueron elevados entre el personal de enfermería que trabajaba en unidades de cuidados intensivos, entre quienes no habían elegido voluntariamente la profesión y entre quienes informaron consumo de alcohol. Los entornos de cuidados intensivos se caracterizan intrínsecamente por la exposición repetida a pacientes en estado crítico, dilemas éticos complejos, altas exigencias emocionales y encuentros frecuentes con el sufrimiento y la muerte, factores que aumentan sustancialmente la vulnerabilidad al agotamiento emocional y al estrés traumático secundario^(13)^. Revisiones recientes han destacado de manera similar que la intensidad de la carga de trabajo, la escasez sistémica de personal, el apoyo gerencial insuficiente y la exposición traumática acumulada son factores principales que contribuyen a la fatiga por compasión y al agotamiento profesional entre el personal de cuidados intensivos^(14,15,24)^. Además, las estrategias de afrontamiento inadaptadas, como el consumo de alcohol, y un bajo compromiso profesional pueden socavar la resiliencia psicológica, acelerando así la progresión de la fatiga por compasión con el tiempo. Estos hallazgos resaltan que la fatiga por compasión va más allá de una vulnerabilidad psicológica individual; más bien, representa un riesgo multifacético para la salud ocupacional determinado por la compleja interacción entre las características personales y los factores sistémicos del lugar de trabajo.

Los análisis de correlación y regresión demostraron que tanto la atención plena como la autocompasión se asociaron significativamente con menores niveles de fatiga por compasión, emergiendo como predictores independientes sólidos en el modelo multivariante final. Estos resultados concuerdan estrechamente con los marcos ocupacionales establecidos que indican que la atención plena y la autocompasión mitigan sistemáticamente el agotamiento profesional, el agotamiento emocional y el estrés traumático secundario entre los profesionales de la salud de primera línea^(7,10)^. Evidencia empírica reciente ha validado de manera similar que unas capacidades superiores de atención plena se corresponden directamente con una menor fatiga por compasión en diversos grupos de personal de enfermería^(7,11,14,25)^. Desde el punto de vista del mecanismo de acción, nuestro modelo predictivo sugiere que las enfermeras que mantienen una atención deliberada hacia las experiencias del momento presente, al tiempo que fomentan actitudes compasivas hacia sí mismas, están sustancialmente mejor preparadas para lidiar con las exigencias clínicas emocionalmente agotadoras. Este fenómeno concuerda plenamente con los marcos contemporáneos de reducción del estrés que enfatizan la doble importancia de la regulación emocional y la flexibilidad cognitiva; dentro de estos modelos, la atención plena facilita la conciencia cognitiva y la aceptación de los estados internos angustiosos, mientras que la autocompasión promueve una respuesta adaptativa y libre de juicios ante los factores estresantes ocupacionales agudos^(4,7)^. Al mismo tiempo, estos recursos psicológicos funcionan de manera altamente complementaria y sinérgica, reponiendo eficazmente las reservas emocionales y fortaleciendo la resiliencia profesional a largo plazo entre las enfermeras clínicas.

Según los modelos de regresión lineal múltiple, la atención plena y la autocompasión explicaron conjuntamente el 21,1 % de la varianza en la fatiga por compasión. Si bien este poder predictivo subraya la importancia crítica de los recursos psicológicos individuales, la varianza restante demuestra implícitamente que la fatiga por compasión es un fenómeno altamente multidimensional regido por dinámicas organizativas y ambientales externas. La evidencia empírica confirma que factores estructurales, como la carga de trabajo excesiva, las condiciones inadecuadas de dotación de personal, los estilos de liderazgo, el apoyo organizacional percibido y el clima ético institucional, determinan en gran medida la trayectoria de la fatiga por compasión y el agotamiento profesional entre el personal de enfermería^(13,14,15,26)^. Por lo tanto, las investigaciones futuras deberían incorporar sistemáticamente estas variables institucionales para construir modelos explicativos más integrados y exhaustivos. Además, se justifican diseños longitudinales y basados en intervenciones para dilucidar las vías temporales y causales precisas que subyacen a estas interacciones. Evidencias recientes indican que los protocolos estructurados basados en la atención plena representan estrategias viables, escalables y altamente reproducibles para fortalecer la resiliencia psicológica, optimizar la calidad de vida profesional y garantizar un bienestar laboral integral en los sistemas modernos de prestación de servicios de salud^(9,19,27,28)^.

## CONCLUSIÓN

Los hallazgos de este estudio subrayan que la atención plena y la autocompasión constituyen activos psicosociales fundamentales que predicen de manera activa niveles más bajos de fatiga por compasión entre el personal de enfermería clínica. Al reforzar la regulación emocional y la bondad hacia uno mismo, estos recursos psicológicos modificables ofrecen una protección sólida contra el agotamiento emocional inherente a los entornos de atención de alta complejidad. La integración de protocolos basados en la atención plena y la autocompasión en las estrategias institucionales de salud tiene el potencial de optimizar simultáneamente el bienestar del personal de enfermería y fortalecer la sostenibilidad organizacional. Las investigaciones futuras deberían aprovechar los diseños longitudinales para dar seguimiento a la eficacia a largo plazo de estas intervenciones e investigar sistemáticamente su interacción sinérgica con variables organizativas sistémicas, tales como la carga de trabajo, los índices de dotación de personal y los climas institucionales.

### Fortalezas y Limitaciones

Una de las principales fortalezas de esta investigación radica en su evaluación específica de constructos psicológicos internos y modificables, específicamente la atención plena y la autocompasión, como contramedidas estratégicas contra la fatiga por compasión en el personal de enfermería. Además, el uso de instrumentos validados psicométricamente y el hecho de haber logrado una tasa de respuesta institucional excepcionalmente alta entre el personal de enfermería clínica en ejercicio activo refuerzan la solidez empírica de nuestros modelos.

No obstante, deben reconocerse varias limitaciones. La naturaleza transversal del estudio excluye por completo cualquier interpretación causal definitiva con respecto a las trayectorias observadas. Además, la omisión de covariables organizacionales estructurales, como las métricas objetivas de la carga de trabajo y los sistemas de apoyo gerencial, puede haber limitado el alcance explicativo del modelo de regresión. Por último, dado que los datos del estudio se recopilaron en un único centro de salud público mediante un enfoque de muestreo por conveniencia no probabilístico, y considerando el predominio demográfico de profesionales relativamente jóvenes y en las primeras etapas de su carrera dentro de la muestra, se debe actuar con cautela al generalizar estas conclusiones a poblaciones de enfermería más amplias o culturalmente diversas.

### Implicaciones para la Práctica

Nuestra evidencia empírica indica que cultivar la atención plena y la autocompasión ofrece una vía eficaz y proactiva para mitigar la fatiga por compasión y reforzar la resiliencia emocional entre el personal de enfermería de primera línea. Desde una perspectiva institucional, incorporar capacitación estructurada en atención plena y talleres de autocompasión en los marcos formales de bienestar laboral ofrece un mecanismo práctico y escalable para abordar de manera preventiva el estrés laboral. Se recomienda encarecidamente a los gerentes de enfermería y a los administradores de la atención de salud que integren estas competencias psicológicas directamente en los protocolos de orientación para enfermeras recién contratadas, en los planes de estudio de capacitación continua en el servicio y en los sistemas de apoyo al personal profesional. A nivel de políticas macro, reconocer los fortificadores psicosociales internos como componentes esenciales de estrategias integrales de salud y seguridad ocupacional es vital para estabilizar la fuerza laboral de enfermería, reducir la intención de rotación y, en última instancia, elevar la seguridad y la calidad de la atención prestada a los pacientes.

## Data Availability

Los datos en los que se basa este artículo se compartirán previa solicitud razonable dirigida al autor correspondiente.
